# Updating our understanding of health-related quality of life issues in children with cancer: a systematic review of patient-reported outcome measures and qualitative studies

**DOI:** 10.1007/s11136-022-03259-z

**Published:** 2022-09-24

**Authors:** Maria Rothmund, Samantha Sodergren, Gudrun Rohde, Teresa de Rojas, Gloria Paratico, Giorgia Albini, Johanna Mur, Anne-Sophie Darlington, Alessandra Majorana, David Riedl, the EORTC Quality of Life Group

**Affiliations:** 1 Division of Psychiatry II, Department of Psychiatry, Psychotherapy, Psychosomatics, and Medical Psychology, Medical University Innsbruck, Innsbruck, Austria; 2 Institute of Psychology, University of Innsbruck, Innsbruck, Austria; 3 School of Health Sciences, University of Southampton, Southampton, UK; 4 Department of Clinical Research, Faculty of Health and Sport Sciences, Kristiansand and Sorlandet Hospital, University of Agder, Kristiansand, Norway; 5 Division of Psychiatry, Marie Curie Palliative Care Research Department, University College London (UCL), London, UK; 6 ACCELERATE, Europe, Brussels, Belgium; 7 Department of Oral Medicine and Paediatric Dentistry, University of Brescia, Brescia, Italy; 8 Ludwig Boltzmann Institute for Rehabilitation Research, Vienna, Austria

**Keywords:** Health-related quality of life, Quality of life, Pediatric oncology, Children with cancer, Concept elicitation

## Abstract

**Background:**

Health-related quality of life (HRQOL) is a key concept in pediatric oncology. This systematic review aims to update the conceptual HRQOL model by Anthony et al. (Qual Life Res 23(3):771–789, 2014), covering physical, emotional, social and general HRQOL aspects, and to present a comprehensive overview of age- and disease-specific HRQOL issues in children with cancer.

**Methods:**

Medline, PsychINFO, the Cochrane Database for Systematic Reviews (CDSR), and the COSMIN database were searched (up to 31.12.2020) for publications using patient-reported outcome measures (PROMs) and qualitative studies in children with cancer (8–14-year) or their parents. Items and quotations were extracted and mapped onto the conceptual model for HRQOL in children with cancer mentioned above.

**Results:**

Of 2038 identified studies, 221 were included for data extraction. We identified 96 PROMS with 2641 items and extracted 798 quotations from 45 qualitative studies. Most items and quotations (94.8%) could be mapped onto the conceptual model. However, some adaptations were made and the model was complemented by (sub)domains for ‘treatment burden’, ‘treatment involvement’, and ‘financial issues’. Physical and psychological aspects were more frequently covered than social issues.

**Discussion:**

This review provides a comprehensive overview of HRQOL issues for children with cancer. Our findings mostly support the HRQOL model by Anthony et al. (Qual Life Res 23(3):771–789, 2014), but some adaptations are suggested. This review may be considered a starting point for a refinement of our understanding of HRQOL in children with cancer. Further qualitative research will help to evaluate the comprehensiveness of the HRQOL model and the relevance of the issues it encompasses.

**Supplementary Information:**

The online version contains supplementary material available at 10.1007/s11136-022-03259-z.

## Plain English summary

Due to improved treatment options, more and more children with cancer survive their disease. However, disease and treatment are still burdensome. This does concern children’s physical health, but also their emotional well-being and social life (e.g., family, friends, school). Thus, the focus shifts from survival to children’s health-related quality of life (HRQOL). Several questionnaires—so-called patient-reported outcome measures (PROMs)—have been developed to assess HRQOL from children’s perspective, but they cover different contents. This indicates that there is no consensus about which issues are relevant for HRQOL in children with cancer. In our study, we systematically investigated what existing PROMs assess as well as which issues were discussed in interview studies with children with cancer or their parents. We then compared our findings with an existing model of HRQOL. Our results widely support this previous model, but we suggest some adaptations: We introduced new subdomains for treatment-related emotional burdens (e.g., fear of needles) and treatment involvement (e.g., shared decision-making). Furthermore, we included financial issues, which were not covered within the previous model. While our study gives a very comprehensive overview of what issues are investigated in children with cancer, we cannot make assumptions about which issues are more or less important. Further interview studies with children are needed to learn more about the importance and understandability of the identified issues. With our review, we want to provide a starting point for the further refinement of our understanding of HRQOL in children with cancer.

## Background

Children are affected by different types of cancer compared with adults and represent a unique patient population with distinct features in terms of physiological and cognitive development. While survival rates have consistently improved over the last decades [1–3], children with cancer still face substantial symptoms and side-effects of the disease and treatment. This may include physical, emotional, and psychosocial, but also school-related aspects of health-related quality of life (HRQOL) [4]. Since most HRQOL aspects are only accessible from the individual's perspective, both the Food and Drug Administration (FDA) and the European Medicines Agency (EMA) recommend the use of patient-reported outcome (PRO) in pediatric oncology [5, 6].

The taskforce for PRO assessment in children and adolescents within the International Society for Pharmacoeconomics and Outcomes Research (ISPOR) has highlighted the importance of age-appropriate assessment [7]: While by the age of 5 years children are able to give basic self-reports, this ability substantially improves by the age of 8 years. From 8 years on, children’s self-report should be considered the most important source of information [8]. However, due to their developmental stage, age-appropriate instruments are needed [8, 9]. At the age of 15, adolescents can complete adult forms [10], but age-specific PROMs are currently being developed to cover the unique challenges at the transition to adulthood [11–15].

Despite the increased awareness of the importance of HRQOL in children with cancer, PROMs are still rarely used in clinical trials [16, 17]. While a generic Standard Set for Pediatric Health Assessment has recently been published [18], no cancer-specific core outcome set has yet been defined. Available HRQOL instruments differ considerably in terms of content [19] and have been criticized for mostly focusing on negative aspects (i.e., impairments instead of functional ability) [20]. While this might be the main focus of clinicians who aim to relieve patients from impairments, children place a high priority on social participation and resources [21]. This might be due to the fact that most questionnaires have either been developed without sufficient patient involvement [22, 23] or developed and validated with adult patients [24]. This is problematic since the involvement of children is a key requirement to ensure content validity of pediatric PRO measures [7].

The most comprehensive conceptual framework for HRQOL in children with cancer so far has been presented by Anthony et al. [25]. In their systematic review, they identified four major HRQOL domains: physical health covers physical functioning and symptoms, while the psychological domain encompasses emotional distress, positive psychological functioning, self-esteem, body image, behavior, and cognitive health. The social domain contains social functioning and relationships, and a general health domain covers the general perception and appraisal of the health status.

The present systematic review builds on the conceptual model of HRQOL in children with cancer provided by Anthony et al. [25] and presents a comprehensive overview of age- and disease-specific HRQOL issues in children with cancer aged 8 to 14 years. To do so, pediatric PROMs as well as qualitative studies with children or their parents are investigated.

## Methods

Following the ISPOR guidelines [7], we focused on a specific predefined age-group. The age-range of 8 to 14 years was determined based on cognitive and social aspects as described above. The review forms part of a larger program of work involving the development of a new questionnaire and follows PROM development guidelines by the Quality of Life Group of the European Organization for Research and Treatment of Cancer (EORTC QLG) [26]. The review was not pre-registered since the most commonly used platforms (e.g., PROSPERO) only accepted COVID-19 related registrations at that point in time. Additional information on the data collection (i.e., template data collection forms; data extracted from included studies; data used for all analyses; other materials used in the review) can be requested from the corresponding author. Where applicable, the results were reported in line with the recommendations of the preferred reporting items of systematic reviews and meta-analyses (PRISMA) guidelines [27] (Supplement 1).

### Search strategy and study selection

In December 2020, a systematic literature search was conducted in several databases: The strategy (see Table 1) used medical subject headings (MeSH terms) to search MEDLINE via PubMed. For PsycINFO and CDSR the search was based on entry terms of these MeSH terms. Corresponding filters were applied to search the COSMIN database of systematic reviews of outcome measurement instruments. The results on PubMed and PsycINFO were additionally filtered for peer-reviewed manuscripts in English, German, French, or Spanish, to match the language skills of the reviewers.

**Table 1:** Search strategy

	Area	MeSH terms for medline via PubMed	Search term for PsycINFO/CDSR^a^	Filters used in the COSMIN database^c^
	Cancer	Neoplasms [MeSH] OR Medical Oncology [MeSH]	Neoplas* OR cancer* OR malignan* OR tumor* OR tumour* OR oncolog* OR carcinom*^b^	Disease: Neoplasms and related symptoms
AND	Age-group	(Child [MeSH] OR Adolescent [MesH] OR Pediatrics [MeSH]) NOT Adult [MeSH]	(child* OR adolescen* OR pediatric* OR paediatric*) NOT adult*	Age: Children (0–18)
AND	HRQOL issues	Health status indicators [MeSH] OR Quality of Life [MeSH]	Quality of life OR QOL OR health-related quality of life OR HRQOL OR health related quality of life OR health status OR health level OR well-being^b^ OR well-being^b^ OR side effect*^b^ OR distress^b^ OR symptom*^b^	
AND	Methods	Patient Outcome Assessment [MeSH] OR Self-Report [MeSH] OR Self-Assessment [MeSH] OR Patient reported outcome measures [MeSH] OR Qualitative Research [MeSH] OR Interview, Psychological [MeSH] OR Surveys and Questionnaires [MeSH]	Patient-centered* OR patient centered* OR patient outcome* OR patient report* OR patient-report* OR self-report* OR self-report* OR qualitative research OR interview* OR survey* OR questionnaire* OR respondent*	Type of measurement: Questionnaires/Interviews/Diaries/Clinical Rating Scales

^a^Based on entry terms for MeSH-terms used to search Medline via PubMed

^b^Additional terms, not represented in entry terms

^c^COSMIN database of systematic reviews of outcome measurement instruments

Studies were selected in a stepwise approach. In each step, teams of at least two reviewers rated eligible papers independently against predefined inclusion and exclusion criteria (see below). First, eligible papers were identified based on their title and abstract by two pairs of reviewers (DR & MR; GR & SS). Second, the full texts of studies identified as relevant by at least one reviewer were re-evaluated in detail by three teams of reviewers [DR & MR; GR & SS; TdR & GP & GA]. The RAYYAN software [28] was used to record these ratings.

### Inclusion and exclusion criteria

Quantitative and qualitative publications were included if full-text papers could be accessed and provided information on HRQOL issues of children with cancer at diagnosis or undergoing curative or palliative treatment between the age of 8 to 14 years. Studies assessing parent- or proxy-report alongside self-report by children were accepted. Studies conducted exclusively in parent samples were only included if parents provided qualitative information on the HRQOL of their child diagnosed with cancer. Studies with a broader age-range including older adolescents or younger children were included if they contained children between 8 and 14 years. Reviews were included for descriptive and cross-referencing purposes only.

Studies were excluded if they *exclusively* (a) investigated healthy or non-cancer samples (mixed samples with cancer patients were included); (b) investigated cancer survivors after treatment completion; (c) consisted of children younger than 8 years (i.e., upper age limit < 8 years); (d) included adolescents and/or adults older than 14 years (i.e., lower age limit > 14 years); or (e) they did not assess HRQOL issues of the children with cancer (e.g., studies focusing on HRQOL of their parents/caregivers, siblings or HCPs; studies relying exclusively on biomarkers, observation- or performance-based clinician reports, or proxy ratings of PROMs). We excluded individual case reports, conference abstracts, and study protocols.

### Data extraction

Data were extracted for quantitative and qualitative studies separately. For quantitative studies, a list of PROMs administered to children with cancer was extracted. To ensure that all relevant PROMs were included, the list was cross-checked against a list of 112 measures collected for the development of the ICHOM Standard Set for Pediatric Health Assessment [18]. Subsequently, a comprehensive list of items from all PROMs was extracted [MR]. If the identified studies did not offer sufficient details and no review copy could be found, authors were contacted.

For qualitative studies, a comparable list was extracted, containing direct quotations from children with cancer or corresponding parents [JM]. These quotations consisted of single sentences or short paragraphs representing children’s or parents’ statements in direct speech in order to provide examples for specific subjects discussed in the qualitative studies.

### Mapping procedure and analysis

All extracted items and quotations were mapped onto the conceptual model of Anthony et al. [25], using an Excel sheet. Each item/quotation was categorized by two reviewers independently [items: DR & MR; GR & SS; TdR & GP & GA; quotations: JM & MR & DR] as representing a domain, subdomain and identifying concept. In case of conflicts, a third party was consulted, and issues were discussed until consensus was reached. Since some items and quotations could relate to more than one domain, subdomain, or identifying concept, raters were provided with basic mapping rules (Supplement 2) and a list of definitions to optimize interrater reliability. Most definitions were drawn from the Encyclopedia of Quality of Life and Well-Being Research [29]. If items did not fit into the existing model, new identifying concepts or subdomains were formulated. Quantitative analysis was based on descriptive statistics.

## Results

**Fig. 1:**
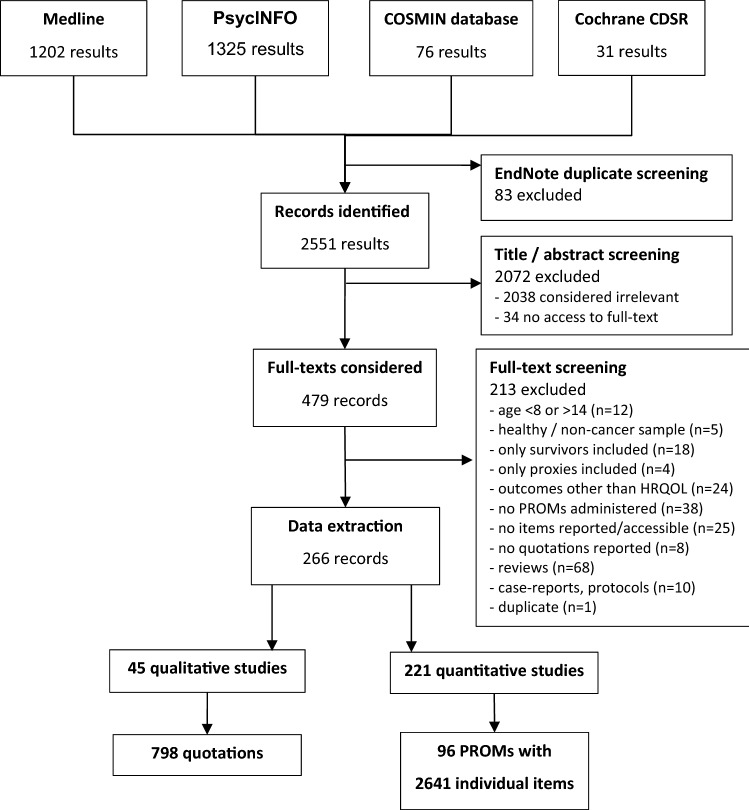
Flowchart of the study selection and data extraction procedure

The initial literature search resulted in a total of 2551 hits. After the removal of 83 duplicates, another 2038 studies were excluded based on their title or abstract and 34 studies were excluded because full texts were not accessible. During the full-text screening of the remaining 479 studies, another 213 studies were excluded, resulting in a total of 266 studies which were included in the analysis. Among them were 221 quantitative studies using 96 different PROMs and 45 qualitative studies. For details refer to Fig. 1.

### Description of questionnaires administered in quantitative studies

Among the 96 included PROMs, only 19 PROMs were cancer-specific. The most frequently used questionnaires were the PedsQL generic core (in 58/221 studies, 26.2%), PedsQL cancer module (37/221, 16.7%), and the Children's Depression Inventory (CDI; 27/221, 12.2%). The complete list of questionnaires is available in Supplement 3.

About a third of the questionnaires (32/96, 33.3%) assessed multidimensional aspects of HRQOL (i.e., physical and psychosocial factors), while the remaining questionnaires focused solely on either psychological (34/96, 35.4%), physical (26/96, 27.1%), or social factors (4/96, 4.2%).

From all PROMs, a pool of 2,682 individual items was extracted. After excluding conditional, determinant, and open-ended questions, 2,641 items were mapped onto the model of Anthony et al. [25]. As presented in Table 2, almost half of these items were assigned to psychological health-aspects (1239/2641, 46.9%), with most items assessing symptoms of emotional distress. Another third of all items covered physical health (862/2641, 32.6%), while social health (463/2641, 17.2%) accounted for nearly one-fifth. General health perception (73/2641, 2.8%) and financial issues (4/2641, 0.2%) were least frequently assessed.

**Table 2:** Number and proportion of items and quotations per domain and subdomains

	Items extracted from PROMs		Quotations from qualitative studies		Total (items and quotations)	
Domains/subdomains	*N*	%	*N*	%	*N*	%
**Total**	**2641**	**100.0**	**798**	**100.0**	**3439**	**100.0**
**Physical Health**	**862**	**32.6**	**340**	**42.6**	**1202**	**35.0**
Symptoms (e.g., fatigue, pain, etc.)	585	67.9	274	80.6	859	71.5
Physical functioning (e.g., mobility, dexterity, etc.)	277	32.1	66	19.4	343	28.5
**Psychological Health**	**1239**	**46.9**	**260**	**32.6**	**1499**	**43.6**
Behavioral (e.g., aggressive, withdrawal, coping, etc.)	145	11.7	47	18.1	192	12.8
Cognitive functioning (e.g., attention, remembering, etc.)	114	9.2	3	1.2	117	7.8
Emotional distress (e.g., afraid, angry, sad, etc.)	564	45.5	68	26.2	632	42.2
Positive psychological functioning (e.g., benefit finding, locus of control, etc.)	201	16.2	73	28.1	274	18.3
Self-esteem ^a^ (e.g., feeling good about self, abilities, body, etc.)	191	15.4	29	11.2	220	14.7
Treatment burden ^b^ (e.g., bothered, procedural anxiety, etc.)	24	1.9	40	15.4	64	4.3
**Social Health**	**463**	**17.5**	**170**	**21.3**	**633**	**18.4**
Relationships (e.g., autonomy, school functioning, etc.)	248	53.6	69	40.6	317	50.1
Social functioning (e.g., with peers, siblings, family, etc.)	151	32.6	57	33.5	208	32.9
Treatment involvement ^b^ (e.g., involvement, shared decision-making, etc.)	64	13.8	44	25.9	108	17.1
**General Health**	**73**	**2.8**	**24**	**3.0**	**97**	**2.8**
**Financial Issues**	4	0.2	4	0.5	8	0.2

Subdomains are displayed in bold

^a^This subdomain also contains ‘body image’, which was a separate subdomain in the conceptual model by Anthony et al. [25]

^b^New subdomain

### Description of qualitative studies

Of the 45 qualitative studies, the majority (24/45; 53.3%) focused on more than one domain, while nine studies (9/45, 20.0%) solely focused on physical aspects, eight (8/45, 15.56%) on psychological health, and four studies on social issues. From all qualitative studies, a total of 798 quotations was extracted and mapped onto the conceptual model by Anthony et al. [25]. As shown in Table 2, most quotations were about physical (340/798, 42.6%) and psychological aspects of HRQOL (260/798, 32.6%), while social health (170/798, 21.3%) and general health issues (24/798, 3.0%) were less frequently discussed in the qualitative studies. Only four quotations concerned financial issues (4/798, 0.5%).

### Updated conceptual model

**Fig. 2:**
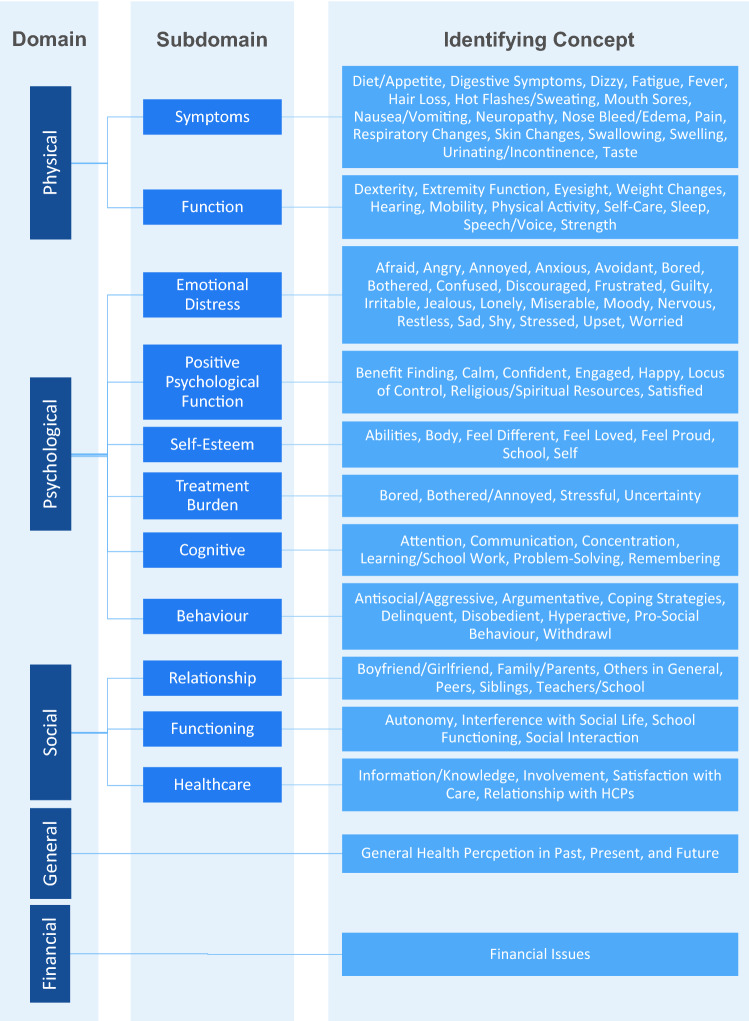
Updated Model of Health-Related Quality of Life Issues in Children with Cancer

Most of the identified items and quotations (3259/3439; 94.8%) were assigned to one of the domains and subdomains of the conceptual model proposed by Anthony et al. [25]. The remaining 180 items and quotations (180/3439, 5.2%) were assigned to three newly introduced (sub)domains. In the psychological domain, we made a new differentiation between ‘emotional distress’ in general and ‘treatment burden’, which encompasses distress explicitly related to the treatment (e.g., procedural anxiety). Furthermore, we incorporated the previously independent subdomain ‘body image’ as an identifying concept into the ‘self-esteem’ subdomain. Within the social domain, the subdomain ‘treatment involvement’ was added. A new domain for ‘financial issues’ was introduced. An overview of the resulting updated model is presented in Table 2 and Fig. 2. All domains and subdomains are described in more detail in the following paragraphs.

The domain *physical health* was defined as ‘*the extent to which objective physical human states are fulfilled in relation to personal or group perceptions of subjective physical well-being*’ [30]. The domain included *physical symptoms* which were represented by 18 different identifying concepts, such as fatigue, fever, hair loss, or pain. The second subdomain was *physical functions*, which was represented by 14 identifying concepts, including mobility, physical activity, or physical strength. Most of the issues could be assigned to one of the two subdomains. However, in the case of some issues (e.g., sleep functioning/insomnia or appetite/taste) the wording of the issue informed whether if it was categorized as a symptom or function. To avoid having similar identifying concepts in different subdomains, a consensus was reached among the researchers and issues were assigned to the subdomain they primarily belonged to.

The domain *psychological health* included a total of six subdomains, namely emotional distress, positive psychological function, self-esteem, treatment burden, cognitive function, and behavior. Most of the items and quotations in this domain could be assigned to the subdomain ‘*emotional distress’* (632/1499, 43.6%) which included negative emotional states such as anxiety, depression, stress, sadness, worry, guilt, shame, anger, and envy. While the assignment to this subdomain was clear, the identifying concepts showed a substantial conceptual overlap (e.g., feeling angry, bothered, annoyed, or frustrated).

The newly introduced subdomain ‘*treatment burden*’ is closely related to emotional distress but covers psychological issues explicitly associated with the treatment. This may include aspects such as procedural anxiety, but also hating to take one's medicine or being bored if the treatment takes a long time. These issues were covered in only six of the identified questionnaires (6/96, 6.3%; SQOLPOP, DISABKIDS DCGM 12/37, PedsQL Child—Transplant, PedsQL Child—SCT, RSQ-PC, USK) and accounted for only 1.9% (24/1239) of items assigned to the psychological domain. In the qualitative studies, however, treatment burden accounted for 15.4% (40/260) of all quotations assigned to the psychological domain and was covered in every fourth study (12/45) [31–42]. Children for example told the interviewer that *‘[…] when the nurse told me that I would have to undergo LP, I thought that it is a surgery, so I was really scared*’ [38] that they were ‘*afraid of unsuccessful treatment*’ [42] or ‘*fed up […] with medication and chemo[-therapy] I have to undergo*’ [34].

The subdomain ‘*positive psychological functions*’ was defined as abilities to successfully adapt and endure under adverse circumstances as well as efficiently recover from subsequent harmful effects [43]. This subdomain consisted of nine functions and positive emotional states, namely benefit finding, locus of control, feeling calm, confident, engaged, happy, responsive, or satisfied and being religious or having spiritual resources.

The subdomain ‘*self-esteem*’ was merged with the previously independent subdomain ‘body image’ due to the overlap of content. We defined ‘self-esteem’ as an evaluative aspect of the self-concept that corresponds with an overall view of the self as worthy or unworthy and the assessment of how people feel in relation to their social standing, their physical appearance or their job or school performance [44]. In our review, this included eight identifying concepts, namely feeling different, loved, or proud, feeling good about self or ones’ abilities, friends and school as well as positive feelings about ones’ personal appearance and physical development.

‘*Cognitive functions*’ included all mental processes to acquire and process knowledge, information, and reasoning [45]. We identified six functions, namely attention, communication, concentration, learning and school-work, problem-solving, and remembering.

In the subdomain ‘*behavior*’ we included all positive and negative active interactions between the individual and their environment. In the review we were able to identify eight—mainly negative—forms of behavior, namely behaving antisocial/aggressive, argumentative, delinquent, disobedient, hyperactive, or withdrawing. Positive forms of behavior included pro-social behavior and active coping strategies.

The domain ‘*social health*’ consisted of three subdomains. *Social functioning* was defined as the ‘ability to achieve personal goals in social interaction while simultaneously maintaining positive relationships’ [46] and included constructs like perceived autonomy and social interactions but also interference with social life due to the disease. *Social relationship* on the other hand describes the ‘network of social resources that an individual perceives’ [47] and was defined as the quality of social relations with family, parents, siblings but also peers, romantic partners, teachers or others in general.

The third subscale ‘*treatment involvement*’ was newly introduced to the model based on our findings. This subscale included social and communicative aspects closely related to the treatment, such as information and knowledge about the disease/treatment, involvement in treatment decisions, satisfaction with care but also the quality of relationship with healthcare professionals. Aspects of treatment involvement were covered in 12 questionnaires (12/96, 12.5%; R-PIE, USK, CUIS, QOLCC, LSS-C, CICS, PAC-QOL, PedsQL Child—cancer/transplant module, TQPM, DISABKIDS DCGM, MANE) and accounted for 13.8% (64/463) of items on social health. The focus on this subscale was even more pronounced in qualitative studies: every fourth quotation addressing social health related to treatment involvement (44/170, 25.9%) and the topic was raised in 17 of the 45 (37.8%) qualitative studies [32, 34, 36, 38–41, 48–57]. One child, for example, explained the need for information by stating ‘The doctor explains each procedure that I have to undergo, everything that I have to go through. I prefer knowing what's going to happen so that I can prepare myself’ [50]. Children also reported lack of involvement in shared decision-making, for example ‘*My mother did [decide when I should train]. Yeah, she decides just about everything*’ [36].

The fourth domain ‘*general health*’ consists of only one subdomain, ‘*general health perception*’. It covers personal views on patients’ own health, sickness, or HRQOL in the past, present, or future.

A new fifth domain was added to incorporate ‘*financial issues*’. This was a less frequent issue, only covered in two questionnaires (EORTC QLQ-C30, KIDSCREEN) and two qualitative studies [34, 51]. The EORTC QLQ-C30 item as well as the quotations refer to a financial burden resulting from the condition and/or treatment. The three items of the KIDSCREEN, however, ask whether children have enough money for daily needs and to participate in social activities with peers.

## Discussion

The aim of this review was to give a comprehensive overview of HRQOL issues for children aged 8–14 years undergoing treatment for cancer. We collected over 2500 individual items from nearly 100 questionnaires used in quantitative studies as well as almost 800 quotations from qualitative studies. Most items and quotations could be assigned to one of the domains and subdomains of the conceptual model of HRQOL in children with cancer provided by Anthony et al. [25]. Thus, the present review widely supports their HRQOL model. The four major domains physical, emotional, social, and general health as well as most subdomains were maintained. However, based on our results, we suggest complementing and clarifying the model.

### Proposed changes to the HRQOL model

Of four proposed changes, two were minor readjustments. We proposed subsuming the previous subdomain ‘body image’ as an identifying concept under the subdomain ‘self-esteem.’ This decision is based on our definition because we regard self-esteem as an evaluative aspect of the self, which also contains feelings towards one’s personal appearance and physical development. Finally, we also identified items and quotations on ‘financial issues,’ which could not be accommodated by the existing model and were therefore assigned to a new, separate domain.

More significant changes were made in the social and psychological domains, where explicitly treatment-related subdomains were introduced. In the psychological domain, a new differentiation was made between general ‘emotional distress’ and ‘treatment burden,’ which covers distress explicitly related to the treatment, as procedural anxiety for instance. One might argue that these aspects could be subsumed under emotional distress and only few questionnaires make this differentiation. However, a large proportion of statements derived from qualitative studies described distress in a direct relation to the treatment). Furthermore, we argue for a specific treatment-related subdomain, as we aim for a model of HRQOL of children undergoing cancer treatment instead of QOL in general.

Within the social domain, we added a subdomain for ‘treatment involvement,’ which covers children’s involvement in shared decision-making, but also their relationships to healthcare-professionals. We considered the healthcare setting as an especially relevant social environment for children with cancer, which is very distinct from other social contexts like family or peers. Communication in this setting has several functions, spanning from sharing information and enabling self-management, to shared decision-making, to sustaining hope, reducing uncertainty, and supporting emotional health [58]. Thus, the quality of social relationships and communication with healthcare providers, is likely to be associated with HRQOL. For example, reduced illness uncertainty has been shown to correlate with improved HRQOL [59]. Therefore, we believe that a specific subdomain for children’s social involvement in healthcare is justified.

### Further reflections on the HRQOL model

Beyond that, we faced other challenges and had further discussions on the model, which did not result in adaptations. One problem occurred within the physical domain, as the differentiation between physical symptoms and functions was not always as obvious as expected. In some cases, such as pain, the assignment was relatively clear. However, in the case of other issues such as sleep vs. insomnia the assignment largely depended on the individual wording of the item.

Another discussion came up regarding the subdomain ‘behavior’ within psychological health because behavior in general is not an HRQOL issue, but rather a way to display, communicate, and handle HRQOL issues. Most behaviors described in items or quotations are social behaviors (e.g., to withdraw from others), which can be considered as indicators for social relationships and functioning. Others are expressions of emotions (e.g., aggressive behaviors) or ways to handle psychological burdens (e.g., coping strategies). We decided to maintain this subdomain and its identifying concepts, as they might be relevant or useful for developing proxy-measures which should incorporate observable contents.

### Lack of consensus on outcome assessment in children with cancer

Our review also sheds light to a key challenge in HRQOL research in children with cancer. With 96 different PROMs, we have identified a surprisingly large number of PROMs, most of which are not cancer-specific. This might reflect a previously criticized lack of consensus regarding which aspects should be considered essential and thus consistently measured in children with cancer [25, 60]. Recently, Algurén et al. [18] have proposed an Overall Pediatric Health Standard Set (OPH-SS) for children. However, so far, no comparable outcome set has been defined specifically for children with cancer. While generic outcome sets and instruments offer the advantage of comparing the results with healthy children or across different diagnoses, they are not tailored to specific problems children with cancer face. Thus, using generic tools includes the risk of ignoring or overlooking relevant disease-specific HRQOL issues. Furthermore, studies from adult oncology have found generic instruments to be of limited usefulness for the comparison of different cancer treatments, as those measures are not able to detect small changes in HRQOL [61].

The third most commonly used instrument was the CDI [62], a generic instrument assessing depressive symptoms. Even though HRQOL questionnaires contain subscales on emotional health, they may not provide the same depth of information. Less items are available per subscale in multidimensional instruments and a choice must be made which aspects should be included.

To make sure that the final selection of items covers what matters to children, PROM development guidelines require patient involvement in the concept elicitation and cognitive interviews [7, 8, 26, 63, 64]. Nevertheless, children with cancer have rarely been involved in the development of PROMs [22, 23]. Consequently, there is only scarce evidence for the content validity of most available PROMs [23, 65]. Thus, it is questionable whether they cover children’s priorities. For instance, Anthony et al. [21] have shown that social health is underrepresented in existing questionnaires, even though children rate social aspects as most important. In our review, the social domain was also represented by less items and quotations than physical and psychological health.

### Strengths, limitations, and outlook

Our review offers an extensive overview of HRQOL issues assessed by PROMs and reported in qualitative studies with children with cancer and their parents. The comprehensiveness can be considered a strength but might also be a limitation. Not only for symptoms and physical functions but also for emotional distress, some identifying concepts show overlapping content. As our review is based on the literature only, we cannot make final statements on the comprehensiveness, relevance, or understandability of the model and its issues from children’s perspective.

For example, we added a domain for ‘financial issues’ that were covered in two PROMs and mentioned in two qualitative studies. However, children might view financial issues as not important or relevant to them or they might not have an understanding of the financial impact of cancer. Thus, it is likely that such issues will not form part of the final EORTC QLG children’s questionnaire which we are currently developing. Questions on finances should perhaps be reserved for caregivers to answer given that they carry more financial responsibilities than children themselves.

We are convinced that, for further refinement of the model, patients, caregivers, and clinical experts should be involved. Thus, we are currently conducting qualitative interviews in an international sample of 8- to 14-year-old cancer patients, their caregivers, and healthcare professionals. In these interviews, participants are invited to describe their understanding of HRQOL before commenting on the comprehensiveness of the updated model as well as the relevance and comprehensibility of all issues.

The selection of included studies was limited by the languages spoken within the reviewers’ team. While we were able to cover studies in English, German, and Spanish and assume that these languages account for the vast majority of scientific literature, we cannot rule out that we could have missed out on information in other languages.

## Conclusion

This review presents a comprehensive overview of HRQOL issues derived from quantitative and qualitative studies. While our findings were mostly in line with the previously proposed conceptual model by Anthony et al. [25], we proposed some adaptations. Mainly, we introduced treatment-related subdomains in the social and psychological domains. Further qualitative studies are needed to evaluate the relevance and comprehensibility of all identified HRQOL issues from children’s perspective. This review may be considered a starting point for a refinement of our understanding and concept of HRQOL in children with cancer.

## Supplementary Information

Below is the link to the electronic supplementary material.

Supplementary file1 (DOCX 39 kb)

Supplementary file2 (DOCX 21 kb)

Supplementary file3 (DOCX 31 kb)
