# Supplementary material for: Updating our understanding of health-related quality of life issues in children with cancer: a systematic review of patient-reported outcome measures and qualitative studies
Source: Qual Life Res. 2022 Sep 24;32(4):965–76. doi: 10.1007/s11136-022-03259-z (PMC9510324; doi:10.1007/s11136-022-03259-z)
Supplement: Supplementary file 2 — Supplementary file2 (DOCX 21 kb) [file 11136_2022_3259_MOESM2_ESM.docx]

Rothmund, Sodergren, Rohde, de Rojas, Paratico, Albini, Mur, Darlington, Majorana, Riedl, on behalf of the EORTC Quality of Life Group:
**Updating our Understanding of Health-Related Quality of Life Issues in Children with Cancer: A systematic review of patient-reported outcome measures and qualitative studies.**

Corresponding author: Dr. David Riedl, David.Riedl@i-med.ac.at

**Supplement 2: Categorization Rules**

General Rules

- If the domain and subdomain are clear, but no perfectly matching identifying concept is available, the closest possible option should be selected.
- If items/quotations are more specific than the identifying concept, the closest possible option should be selected.
  e.g., headaches -> Physical – symptom – pain
- If items/quotations cannot be assigned to an identifying concept, the option ‘Other’ should be selected.
  - Items/quotations referring to the financial situation of the children and their families should be categorized as ‘Financial’.
- If an item/quotation covers more than one identifying concept of the same subdomain, the predominant one should be selected.
  e.g. I feel sad, worried or unhappy. -> choose sad
- For possibly ambiguous items/quotations like pain or strength (mental or physical?), the physical domain/subdomain/concept should be selected.
- There are items/quotations on cognitive functioning that don’t refer to one of the specific identifying concepts (i.e., Attention, Learning, Remembering, Problem-Solving etc.), but schoolwork in general. In this case choose “General”.
  e.g., I have trouble with my schoolwork -> choose “General”

Interfering issues

i.e., items/quotations referring to connected aspects, e.g. “I can’t sleep *(=effect)* because I have pain *(=cause)*”

- If the cause is either a symptom, emotional distress or cognitive functioning, the cause should be rated. 
  e.g., I can’t sleep (*=effect*) because I have pain (*=cause*).
  🡪 Cause = physical health – symptom – pain  
  e.g., Is your pain *(=cause)* bothering you *(=effect)*?
  🡪 Cause = physical health – symptom – pain
- If the cause is the disease or treatment in general, the effect should be rated.  
  e.g., Does your health (*=cause*) now limit you in... bending, kneeling, or stooping (*=effect*)?
  🡪 Effect = Physical health – physical function – physical activity

In case of uncertainty

- In a separate column, raters have the option to comment on each item/quotation, to note questions, and to suggest additional categories.
