# Supplementary material for: Updating our understanding of health-related quality of life issues in children with cancer: a systematic review of patient-reported outcome measures and qualitative studies
Source: Qual Life Res. 2022 Sep 24;32(4):965–76. doi: 10.1007/s11136-022-03259-z (PMC9510324; doi:10.1007/s11136-022-03259-z)
Supplement: Supplementary file 3 — Supplementary file3 (DOCX 31 kb) [file 11136_2022_3259_MOESM3_ESM.docx]

Rothmund, Sodergren, Rohde, de Rojas, Paratico, Albini, Mur, Darlington, Majorana, Riedl, on behalf of the EORTC Quality of Life Group:
**Updating our Understanding of Health-Related Quality of Life Issues in Children with Cancer: A systematic review of patient-reported outcome measures and qualitative studies.**

Corresponding author: Dr. David Riedl, David.Riedl@i-med.ac.at

**Supplement 3: Table of identified Patient-Reported Outcome Measures (PROMs), basic main characteristics, and the frequency of their use.**

|  | FULL NAME | AGE SPAN(S) | AGE GROUP | SCOPE | NUMBERS OF STUDIES | CANCER- SPECIFIC |
| --- | --- | --- | --- | --- | --- | --- |
| PedsQL Generic | Pediatric Quality of Life Inventory - Generic Module | 8-12; 13-18 years | child, adolescent | multidimensional (quality of life) | 58 |  |
| PedsQL Cancer | Pediatric Quality of Life Inventory - Cancer Module | 8-12; 13-18 years | child, adolescent | multidimensional (quality of life) | 37 | x |
| CDI | Children's Depression Inventory | 7-17 years | child, adolescent | psychological (depression/sadness) | 27 |  |
| MSAS (7-12 / 10-18) | Memorial Symptom Assessment Scale | 7-12, 10-18 years | child, adolescent | symptom (various) | 24 |  |
| FS-C | Fatigue Scale Childen (FSA for children) | 7-12 years | child | symptom (fatigue) | 14 |  |
| PedsQL Fatigue | Pediatric Quality of Life Inventory - Multidimensional Fatigue Module | 8-12; 13-18 years | child, adolescent | symptom (fatigue) | 14 |  |
| Ped PROMIS - anxiety | Pediatric Patient-Reported Outcomes Measurement Information System – anxiety scale | 8-17 years | child, adolescent | psychological (anxiety/uncertainty) | 13 |  |
| FSA | Adolescent Fatigue Scale | 13-18 years | adolescent | symptom (fatigue) | 12 |  |
| Ped PROMIS - depressive symptoms | Pediatric Patient-Reported Outcomes Measurement Information System – depressive symptoms scale | 8-17 years | child, adolescent | psychological (depression/sadness) | 12 |  |
| Ped PROMIS - fatigue | Pediatric Patient-Reported Outcomes Measurement Information System – fatigue scale | 8-17 years | child, adolescent | symptom (fatigue) | 11 |  |
| RCMAS | Revised Children's Manifest Anxiety Scale | 6-19 years | child, adolescent | psychological (anxiety/uncertainty) | 11 |  |
| CBCL-YSR-11-18 | Child Behavior Checklist  Youth Self-Report version | 11-18 years | adolescent | multidimensional | 10 |  |
| Ped PROMIS - pain interference | Pediatric Patient-Reported Outcomes Measurement Information System – pain interference scale | 8-17 years | child, adolescent | symptom (pain) | 10 |  |
| QOLCC (7-12 / ADO) | Quality of Life in Childhood Cancer | 7-12, 13-18 years | child, adolescent | multidimensional (quality of life) | 9 | x |
| CHQ | Child Health Questionnaire | 5-18 years | child, adolescent | multidimensional | 8 |  |
| Ped PROMIS - mobility | Pediatric Patient-Reported Outcomes Measurement Information System – mobility scale | 8-17 years | child, adolescent | symptom (mobility) | 8 |  |
| Ped PROMIS - peer relationships | Pediatric Patient-Reported Outcomes Measurement Information System – peer relationships scale | 8-17 years | child, adolescent | social | 8 |  |
| UCLA-PTSD-I | University of California Los Angeles Posttraumatic Stress Disorder Reaction Index | <6, 7-18 years | child, adolescent | psychological (other) | 7 |  |
| YLOT | Youth Life Orientation Test | grades 3-6 | child | psychological (other) | 7 |  |
| SDS | Symptom Distress Scale | 18-89 years | adult | multidimensional | 6 |  |
| ChIMES | Children's International Mucositis Evaluation Scale | 0-18 years | child, adolescent | symptom (oral) | 5 |  |
| Ped PROMIS - upper extremity function | Pediatric Patient-Reported Outcomes Measurement Information System – upper extremity function scale | 8-17 years | child, adolescent | symptom (extremity function) | 5 |  |
| DISABKIDS / DCGM | DISABKIDS Chronic Generic Module | 8-16 years | child, adolescent | multidimensional (quality of life) | 4 |  |
| HUI-15Q | Health Utilities Index - self-administered questionnaire | >12 | adolescent, adult | multidimensional | 4 |  |
| OMDQ | Oral Mucositis Daily Questionnaire | >18 years | adult | symptom (oral) | 4 |  |
| Ped PROMIS - anger | Pediatric Patient-Reported Outcomes Measurement Information System – anger scale | 8-17 years | child, adolescent | psychological (other) | 4 |  |
| PedsQL Brain Tumor | Pediatric Quality of Life Inventory – Brain Tumor Module | 8-12; 13-18 years | child, adolescent | multidimensional (quality of life) | 4 | x |
| ASWS | Adolescent Sleep Wake Scale | 12-25 years | adolescent, young adult | symptom (sleep) | 3 |  |
| BSI | Brief Symptom Inventory | >13 years | adolescent, adult | symptom (various) | 3 |  |
| CES-DC | Centre for Epidemiologic Studies Depression Scale for Children | 6-17 years | child, adolescent | psychological (depression/sadness) | 3 |  |
| CRIES / IES | Children's Impact of Events Scale | 8-18 years | child, adolescent | psychological (other) | 3 |  |
| EORTC-QLQ-C30 | European Organization for Research and Treatment of Cancer – Quality of Life Questionnaire – Core (30 items) | >18 years | adult | multidimensional (quality of life) | 3 | x |
| HMAC | Hemmingway Measure of Adolescent Connectedness | 9-18 years,  grade 6-12 | child, adolescent | social | 3 |  |
| KINDL Generic | Revidierter Fragebogen für KINDer und Jugendliche zur Erfassung der gesundheitsbezogenen Lebensqualität, Generisches Hauptinstrument | 4-17 years | child, adolescent | multidimensional (quality of life) | 3 |  |
| MMQL-YF | Minneapolis-Manchester Quality of Life instrument | 8-12 years | child | multidimensional (quality of life) | 3 | x |
| RSES | Rosenberg Self Esteem Scale | high school | adult | psychological (other) | 3 |  |
| AQOL | Adolescent QOL Scale | 9-20 years | child, adolescent, young adult | multidimensional (quality of life) | 2 |  |
| BBSC | Benefit/Burden Scale for Children = 10 BFSC items + 10 new burden items | 8-18 years | child, adolescent | psychological (other) | 2 |  |
| BDI | Beck's Depression Inventory | 18-80 years | adult | psychological (depression/sadness) | 2 |  |
| BFSC | Benefit Finding Scale for Children | 7-18 years | child, adolescent | psychological (other) | 2 |  |
| DSRS / BDS | Depression Self Rating Scale / Birleson Depression Scale | 8-14 years | child, adolescent | psychological (depression/sadness) | 2 |  |
| EQ-5D-Y | EuroQol 5 Dimensions – Youth | 8-15 years | child, adolescent | multidimensional (quality of life) | 2 |  |
| FDI | Functional Disability Inventory | 8-17 years | child, adolescent | symptom (various) | 2 |  |
| KIDSCREEN | KIDSCREEN | 8-18 years | child, adolescent | multidimensional (quality of life) | 2 |  |
| PedsQL PF VAS | Pediatric Quality of Life Inventory – Present Functioning scales  (visual analogue scale) | 5-18 years | child, adolescent | multidimensional (quality of life) | 2 |  |
| RSQ-PC | Responses to Stress Questionnaire - Pediatric Cancer Version | >9 years | child, adolescent, adult | psychological (other) | 2 | x |
| SCARED | Screen for Child Anxiety Related Disorders | 8-18 years | child, adolescent | psychological (anxiety/uncertainty) | 2 |  |
| SDQ | Strength and Difficulties Questionnaire | 3-16 years | child, adolescent | multidimensional | 2 |  |
| SSPedi | Symptom Screening in Pediatrics | 8-18 years | child, adolescent | symptom (various) | 2 | x |
| TRSC-C | Therapy-Related Symptom Checklist Children | 5-11, 12-17 years | child, adolescent | symptom (various) | 2 | x |
| USK | Uncertainty Scale for Kids | 8-18 years | child, adolescent | psychological (anxiety/uncertainty) | 2 |  |
| AQC | Alexithymia Questionnaire for Children | primary-secondary school | child, adolescent | psychological (other) | 1 |  |
| BPI | Brief Pain Inventory | not specified | not specified | symptom (pain) | 1 |  |
| CATIS | Child Attitude Towards Illness Scale | 8-12 years | child | multidimensional | 1 |  |
| CHIP-CE/CRF | Child Health and Illness Profile Child Edition / Child Report Form | 6-11 years | child | multidimensional | 1 |  |
| CHLCS | Children's Health Locus of Control scale (Parcel & Meyer) | 7-12 years | child | psychological (other) | 1 |  |
| CHS | Children's Hope Scale | 8-16 years | child, adolescent | psychological (other) | 1 |  |
| CICS | Child Involvement in Care Scale | 8-12 years | child | social | 1 |  |
| CUIS | Children's Uncertainty in Illness Scale | 8-18 years | child, adolescent | psychological (anxiety/uncertainty) | 1 |  |
| EASI | Emotionality Activity, Sociability and Impulsivity Inventory | not specified | not specified | psychological (other) | 1 |  |
| ESAS | Edmonton Symptom Assessment Scale | >18 years | adult | symptom (various) | 1 |  |
| FACT-E / -ECS | FACT Esophageal Cancer Subscale | >18 years | adult | symptom (various) | 1 | x |
| FACT-G | FACT General | >18 years | adult | multidimensional | 1 | x |
| FoP-Q-SF/C | Fear of Progression Questionnaire for Children | 10-18 years | child, adolescent | psychological (anxiety/uncertainty) | 1 | x |
| HADS | Hospital Anxiety and Depression Scale | >18 years | adult | psychological (anxiety; depression) | 1 |  |
| HHI | Hearth Hope Index | >18 years | adult | psychological (other) | 1 |  |
| ILK | Inventar Lebensqualität Kinder | 6-18 years | child, adolescent | multidimensional (quality of life) | 1 |  |
| KINDL Oncology | Revidierter Fragebogen für KINDer und Jugendliche zur Erfassung der gesundheitsbezogenen Lebensqualität, Krankheitsspezifisches Modul ‘Onkologie’ | 7-17 years | child, adolescent | multidimensional (quality of life) | 1 | x |
| LSS-C | Life Situation Scale for Children | 7-12 years | child | multidimensional | 1 |  |
| MAF | Multidimensional Assessment of Fatigue | 18-64 years | adult | symptom (fatigue) | 1 |  |
| MANE | Morrow Assessment of Nausea and Emetics/Emesis | >18 years | adult | symptom (nausea) | 1 | x |
| MDASI | M.D. Anderson Symptom Inventory | >18 years | adult | symptom (various) | 1 | x |
| OHIP-14 | Oral Health Impact Profile | >18 years | adult | symptom (oral) | 1 |  |
| PAC-QOL | Pediatric Advanced Cancer Quality of Life questionnaire | 2-18 years | child, adolescent | multidimensional (quality of life) | 1 | x |
| PCQ | Procedural Coping Questionnaire | 6-15 years | child, adolescent | psychological (other) | 1 |  |
| PCS-C | Pain Catastrophizing Scale for Children | 8-16 years | child, adolescent | symptom (pain) | 1 |  |
| Ped PRO-CTCAE | Pediatric Patient-Reported Outcome version of the Common Terminology Criteriy for Adverse Events | 7-17 years | child, adolescent | multidimensional | 1 | x |
| Ped PROMIS - pain intensity | Pediatric Patient-Reported Outcomes Measurement Information System – pain intensity scale | 8-17 years | child, adolescent | symptom (pain) | 1 |  |
| Ped PROMIS - psychological stress experiences | Pediatric Patient-Reported Outcomes Measurement Information System – psychological stress experiences scale | 8-17 years | child, adolescent | psychological (other) | 1 |  |
| Peds FAACT | Pediatric Functional Assessment of Anorexia/Cachexia Treatment | 7-12 years | child | multidimensional | 1 | x |
| Peds-FACT-BrS | Peds FACT Brain Tumor Survivor | 7-18 years | child, adolescent | multidimensional | 1 | x |
| PedsQL Transplant | Pediatric Quality of Life Inventory - Transplant Module | 8-12; 13-18 years | child, adolescent | multidimensional (quality of life) | 1 |  |
| PGWB | Psychological General Well-Being Scale | not specified | not specified | psychological (other) | 1 |  |
| Piers-Harris | Piers-Harris Children's self-concept scale | 7-18 years | child, adolescent | psychological (other) | 1 |  |
| PSQ / YPSQ | (Young) Positive Schema Questionnaire | 18-75 years | adult | psychological (other) | 1 |  |
| PSQI | Pittsburgh Sleep Quality Index | >13 years | adolescent, adult | symptom (sleep) | 1 |  |
| PSS | Perceived Stress Scale (Cohen) | >18 years | adult | psychological (other) | 1 |  |
| R-MHLCS | Revised Multidimensional Health Locus of Control Scale | not specified | not specified | psychological (other) | 1 |  |
| RS10 | Resilience Scale for Childen 10 | 7-12 years | child | psychological (other) | 1 |  |
| SAB | Scale of Available Behaviors | grade 3 and higher | child, adolescent | social | 1 |  |
| SF-36 | short form | > 14 years | adolescent, adult | multidimensional | 1 |  |
| SQOLPOP | Scale for Quality of Life in Pediatric Oncology Patients | 13-18 years | adolescent | multidimensional (quality of life) | 1 | x |
| TACQOL / TAAQOL | TNO AZL Children's/Adolescents’ Quality of Life | 5-15, >18 years | child, adolescent, adult | multidimensional (quality of life) | 1 |  |
| TQPM | Total Quality Pain Management Program | 8-12 years | child | symptom (pain) | 1 |  |
| WAI | Weinberger Adjustment Inventory | 10-65 years | child, adolescent, adult | psychological (other) | 1 |  |
| PedsQL SCT | Pediatric Quality of Life Inventory – Stem Cell Transplant Module | 8-12; 13-18 years | child, adolescent | multidimensional (quality of life) | 0* |  |
| * The PedsQL SCT was mentioned in a study without being applied. Nevertheless, it was considered relevant for the present review, as stem cell transplants are a common treatment in pediatric oncology. | | | | | | |
